# Membrane Resonance in Pyramidal and GABAergic Neurons of the Mouse Perirhinal Cortex

**DOI:** 10.3389/fncel.2021.703407

**Published:** 2021-07-22

**Authors:** Noemi Binini, Francesca Talpo, Paolo Spaiardi, Claudia Maniezzi, Matteo Pedrazzoli, Francesca Raffin, Niccolò Mattiello, Antonio N. Castagno, Sergio Masetto, Yuchio Yanagawa, Clayton T. Dickson, Stefano Ramat, Mauro Toselli, Gerardo Rosario Biella

**Affiliations:** ^1^Department of Biology and Biotechnology Lazzaro Spallanzani, University of Pavia, Pavia, Italy; ^2^Department of Brain and Behavioral Sciences, University of Pavia, Pavia, Italy; ^3^Department of Genetic and Behavioral Neuroscience, Gunma University, Maebashi, Japan; ^4^Department of Psychology, University of Alberta, Edmonton, AB, Canada; ^5^Department of Industrial and Information Engineering, University of Pavia, Pavia, Italy

**Keywords:** membrane resonance, pyramidal neuron, GABAergic interneuron, perirhinal cortex, patch-clamp

## Abstract

The perirhinal cortex (PRC) is a polymodal associative region of the temporal lobe that works as a gateway between cortical areas and hippocampus. In recent years, an increasing interest arose in the role played by the PRC in learning and memory processes, such as object recognition memory, in contrast with certain forms of hippocampus-dependent spatial and episodic memory. The integrative properties of the PRC should provide all necessary resources to select and enhance the information to be propagated to and from the hippocampus. Among these properties, we explore in this paper the ability of the PRC neurons to amplify the output voltage to current input at selected frequencies, known as membrane resonance. Within cerebral circuits the resonance of a neuron operates as a filter toward inputs signals at certain frequencies to coordinate network activity in the brain by affecting the rate of neuronal firing and the precision of spike timing. Furthermore, the ability of the PRC neurons to resonate could have a fundamental role in generating subthreshold oscillations and in the selection of cortical inputs directed to the hippocampus. Here, performing whole-cell patch-clamp recordings from perirhinal pyramidal neurons and GABAergic interneurons of GAD67-GFP^+^ mice, we found, for the first time, that the majority of PRC neurons are resonant at their resting potential, with a resonance frequency of 0.5–1.5 Hz at 23°C and of 1.5–2.8 Hz at 36°C. In the presence of ZD7288 (blocker of HCN channels) resonance was abolished in both pyramidal neurons and interneurons, suggesting that I_h_ current is critically involved in resonance generation. Otherwise, application of TTx (voltage-dependent Na^+^ channel blocker) attenuates the resonance in pyramidal neurons but not in interneurons, suggesting that only in pyramidal neurons the persistent sodium current has an amplifying effect. These experimental results have also been confirmed by a computational model. From a functional point of view, the resonance in the PRC would affect the reverberating activity between neocortex and hippocampus, especially during slow wave sleep, and could be involved in the redistribution and strengthening of memory representation in cortical regions.

## Introduction

The perirhinal cortex (PRC) is a polymodal associative ventral region of the temporal lobe located laterally to the rhinal sulcus. It is connected with many sensory and polymodal areas, reward-related cortices, and other structures of the medial temporal lobe (MTL) such as the entorhinal and postrhinal (or parahippocampal in primates) cortices, the amygdala (Pinto et al., 2006; Biella et al., 2010), and the hippocampus (Witter et al., 2000; Kealy and Commins, 2011; Suzuki and Naya, 2014). In recent years, there has been an increasing interest in the role played by the perirhinal cortex in cognitive functions such as declarative learning and memory, as well as interest in its susceptibility during the initial stages of specific neurodegenerative diseases like Alzheimer’s. More specifically, PRC is involved in recognition memory, visual perception, and associative processes. Our current understanding of the role of PRC has been primarily based on lesion experiments, but the underlying functional mechanisms determining how it executes these tasks remain to be uncovered. Based on cytoarchitectonic properties and anatomical connections, PRC should play a crucial role in processing information directed from and to the hippocampal formation. The key role of the perirhinal region, that is not simply a passive relay station, is underscored. Indeed, PRC has a robust inhibitory system (de Curtis and Paré, 2004) that acts as an active gate to selectively allow information to propagate from other cortical regions to the hippocampus and vice versa. In the PRC, in fact, excitatory neocortical inputs undergo a powerful inhibitory block maintained by local GABAergic interneurons (Martina et al., 2001; Biella et al., 2002; Garden et al., 2002; Willems et al., 2018). Moreover, PRC importantly retains integrative properties that are essential for different memory and perceptual tasks (Biella et al., 2001; Murray and Richmond, 2001; Davachi, 2006; Staresina and Davachi, 2008; Suzuki and Naya, 2014).

According to this state of the art, we suggest that the inhibitory barrier in the PRC could allow a selection of relevant inputs directed to and from the hippocampus. To this purpose, namely the ability to select and enhance specific inputs, different cellular and network strategies could be applied, like for example to enhance synaptic plasticity (Perugini et al., 2012) as well as to generate oscillatory patterns in neuronal networks. Furthermore, it has been shown that subpopulations of neurons in many cerebral areas, such as neocortex (Hutcheon et al., 1996, Sun et al., 2014), entorhinal cortex (Lampl and Yarom, 1997; Hutcheon and Yarom, 2000; Haas and White, 2002; Erchova et al., 2004; Engel et al., 2008), and hippocampus (Leung and Yu, 1998; Pike et al., 2000; Hu et al., 2002; Erchova et al., 2004; Narayanan and Johnston, 2007), are able to amplify the output voltage to current input at selected frequencies. This property is called membrane resonance. Within cerebral circuits the resonance of different types of neurons operates as a filter toward inputs signals at certain frequencies, to coordinate network activity in the brain by affecting the rate of neuronal firing (Hutcheon and Yarom, 2000), to influence the precision of spike timing (Desmaisons et al., 1999; Haas and White, 2002; Schaefer et al., 2006), and to cause spike clustering (Chen and Shepherd, 1997; Desmaisons et al., 1999; Pedroarena et al., 1999; Wu et al., 2001; Izhikevich et al., 2003). Resonant behavior emerges as a result of an interplay between membrane passive and active properties due to the frequency-dependent increase of membrane impedance produced by voltage-dependent ion channels (Hutcheon and Yarom, 2000). Several voltage-dependent ion currents are critical for the genesis of the resonance such as M-type potassium current, T-type calcium current, h-type hyperpolarized-activated cationic current, whereas other ones such as the persistent sodium current or the potassium inward rectifier current can act as amplifying currents that facilitate membrane resonance. The ability of perirhinal neurons to resonate could have a fundamental role in generating subthreshold oscillations and in the selection of cortical inputs directed to the hippocampus. At present, there is no report about a possible resonant behavior of neurons located in the PRC.

Shay et al. (2012) demonstrated that, among the different areas of the parahippocampal region, the neurons of the medial entorhinal cortex (EC) show a maximum resonant frequency ranging from 4 to 8 Hz (theta wave) suggesting that these properties could contribute to the generation of the firing dynamics of grid cells in the medial EC that are related to the processing of spatial information. Neurons recorded from the lateral EC that is strongly connected to the PRC (Burke et al., 2018) showed a lower resonant frequency, between 1 and 2 Hz, very similar to that observed in neocortical neurons (0.7–2.5 Hz) (Hutcheon et al., 1996). Furthermore, resonant behavior has been observed in different subregions of the hippocampus. Hippocampal pyramidal neurons showed a resonant frequency ranging among 2–8 Hz (Pike et al., 2000; Hu et al., 2002) whereas two subpopulations of GABAergic interneurons showed either a lower (1–3 Hz, the horizontal interneuron) or higher (10–50 Hz, fast-spiking interneurons) resonant frequency bandwidth. Likely, the resonance properties of neurons and their synaptic interactions within the different areas of the hippocampal region underlie state-dependent network oscillations that may serve as a temporal modality to associate linked and distant neural networks. In this way, by binding different neural networks, resonance could act as a potential cellular mechanism to temporally coordinate information directed to and from the hippocampus.

A systematic study of the resonance properties in the perirhinal neurons has not been performed yet. In this study, we report for the first time that a sizeable subpopulation of both pyramidal cells and GABAergic interneurons of the PRC are able to resonate in a selected range of frequencies (1–2.5 Hz).

## Materials and Methods

### Animals and Brain Slice Preparation

Juvenile (P17–P27) heterozygous GAD67-GFP knock-in mice (Tamamaki et al., 2003) were used for all experiments. Experimental handling of the animals was performed in accordance with EU directive 86/609/EEC, approved by the National Ministry of Health, and designed to minimize the number of the animals and their suffering.

Animals were anesthetized by inhalation of isoflurane and decapitated. The whole brain was removed and submerged in cold (∼4°C) carboxygenated (95% O_2_, 5% CO_2_) cutting solution (Sucrose 70 mM, NaCl 80 mM, KCl 2.5 mM, NaHCO_3_ 26 mM, Glucose 15 mM, MgCl_2_ 7 mM, CaCl_2_ 1 mM, NaH_2_PO_4_ 1.25 mM; pH 7.3). Coronal 350 μm-thick slices containing the rhinal sulcus were prepared using a vibratome (DTK-1000, Dosaka EM). Following cutting, the slices were allowed to equilibrate for at least 1 h in a recovery chamber filled with carboxygenated artificial cerebrospinal fluid (aCSF) medium (NaCl 125 mM, KCl 2.5 mM, NaHCO_3_ 26 mM, Glucose 15 mM, MgCl_2_ 1.3 mM, CaCl_2_ 2.3 mM, NaH_2_PO_4_ 1.25 mM; pH 7.3).

### Electrophysiological Recordings

Recordings were performed at room temperature (∼23°C) on submerged slices perfused at 1.4 ml/min with aCSF, unless otherwise stated. The recording chamber was mounted on an E600FN microscope connected to a near-infrared CCD camera. Data were derived from perirhinal pyramidal neurons and GAD67-GFP-expressing GABAergic interneurons using the whole-cell patch-clamp technique in voltage- and current-clamp modes. Pipettes were produced from borosilicate glass capillary tubes (Hilgenberg GmbH) using a horizontal puller (P-97, Sutter instruments) and filled with the following intracellular solution: K-gluconate 130 mM, NaCl 4 mM, MgCl_2_ 2 mM, EGTA 1 mM, creatine phosphate 5 mM, Na_2_ATP 2 mM, Na_3_GTP 0.3 mM, Hepes 10 mM (pH 7.3 with KOH). Series resistance was minimized and monitored throughout the experiment (R_s_ initial = 9.2 ± 0.3; R_s_ final = 11.7 ± 0.5 for pyramidal neurons, *N* = 128 and R_s_ initial = 11.7 ± 0.5; R_s_ final = 13.8 ± 0.5 for GABAergic interneurons, *N* = 70), however, it was not compensated by using the bridge balance circuit. Recordings were made with a MultiClamp 700B amplifier (Molecular Devices) and digitized with a Digidata 1322 computer interface (Molecular Devices). Data were acquired using the software Clampex 9.2 (Molecular Devices), sampled at 20 kHz, filtered at 10 kHz, and analyzed with the software Clampfit 10.2 (Molecular Devices) and Origin 6.0 (Microcal).

### Morphology of Recorded Neurons

To confirm the identity of the recorded cells by their morphology, biocytin (3 mg/ml, Sigma) was added to the intracellular solution and it diffused into the cell through the patch micropipette during electrophysiological recording. Following recordings slices were fixed in 4% paraformaldehyde for 30 min, washed with PBS (Dulbecco’s phosphate buffer saline, Sigma), then incubated overnight with 10 μg/ml DAPI (4′,6-diamidino-2-phenylindole), and 5 μg/ml Alexa Fluor 568-conjugated streptavidin (Molecular Probes). Next day slices were mounted on microscope slides using DAKO Mounting Medium. Images were acquired by confocal microscopy [microscope Leica TCS SP2 equipped with three laser lines: (i) Ar/UV laser with emissions at 351 and 364 nm; (ii) Ar/Vis laser with emissions at 458 and 488 nm and (iii) HeNe laser with emissions at 543 and 633 nm] using a dedicated acquisition software (LCS software). Cells were reconstructed by using Imaris software (Bitplane).

### Characterization of the Passive and the Firing Properties of the Neurons

For each recorded cell we calculated the membrane capacitance (C_m_), the input resistance (R_in_), and the resting membrane potential (V_r_). C_m_ was estimated by integrating the capacitive current evoked by a −10 mV pulse, whereas R_in_ was calculated from the same protocol at the end of a 20 ms pulse, when the current trace reached the steady state. V_r_ was detected in current-clamp mode with 0pA current injection. To characterize the features of the action potentials (APs), we injected positive supra-threshold current steps. The AP threshold (AP_T__h_) is the value of the membrane potential at which a rapid upstroke of the AP starts (corresponding to the value of potential at which the action potential temporal derivative crosses 20 V/s). The AP amplitude (AP_A_) was measured as the voltage difference between the top of the spike and the AP_Th_. The AP duration (AP_D_) was calculated as the spike width measured at half-maximal spike amplitude. The firing pattern of each cell was categorized through a twofold quantitative analysis. Specifically, we evaluated i) the inter-spike-intervals (ISIs) and ii) the coefficients of variation for a sequence of ISIs (CV2) (Holt et al., 1996; Shinomoto et al., 2009; Komendantov et al., 2019; Graf et al., 2020). ISIs were computed as the temporal separation of two contiguous spikes repeated for each pair of APs of the neuronal discharge. CV2 was calculated as in Holt et al. (1996) by the following Equation (1) applied for all the ISIs of the discharge:

(1) $$\mathrm{CV2}=\frac{2*\left|{\mathrm{ISI}}_{i+1}-{\mathrm{ISI}}_{i}\right|}{{\mathrm{ISI}}_{i+1}+{\mathrm{ISI}}_{i}}$$

### Characterization of Subthreshold Resonance

To characterize the resonant behavior of the cells, the *impedance amplitude profile* (ZAP) method was used (Hutcheon et al., 1996; Hutcheon and Yarom, 2000). A sinusoidal current with constant amplitude (60 pA peak-to-peak) and linearly increasing frequency from 0 to 15 Hz (ZAP current of 50 s) was applied. The protocol was repeated 3–5 times for each cell and the voltage responses were recorded and averaged. Resonance occurs as a peak in the voltage response at a specific frequency (F_res_). In some experiments the reproducibility of the voltage response has been demonstrated by applying modified ZAP current protocols (i.e., ZAP current with a 40 pA or a 20-pA peak-to-peak amplitude, inverted ZAP current with a 15–0 Hz declining frequency, negative ZAP current with the first peak of the stimulus oriented negatively). The impedance profile [Z(f)] was calculated by dividing the Fast Fourier Transform (FFT) of the membrane potential response (V) by the FFT of the ZAP current (I), as indicated in Equation (2).

(2) $$Z\,\left(f\right)=\frac{F\,F\,T\,\left[V\,\left(t\right)\right]}{F\,F\,T\,\left[I\,\left(t\right)\right]}$$

Z(f) is a complex quantity (Z(f) = Z *Real*+ iZ*Imaginary*) that can be plotted as a vector whose magnitude (| Z(f)|) and phase (φ_z_(f)) are given by the expressions (3) and (4), respectively.

(3) $$\left|Z\,\left(f\right)\right|=\sqrt{{\left(Z,R\,e\,a\,l\right)}^{2}+{\left(Z,I\,m\,a\,g\,i\,n\,a\,r\,y\right)}^{2}}$$

(4) $$\phi_{z}\,\left(f\right)=\tan^{-1}\left(\frac{Z,I\,m\,a\,g\,i\,n\,a\,r\,y}{Z,R\,e\,a\,l}\right)$$

The plot of the impedance phase as a function of frequency indicates the phase shift of the voltage wave relative to the current wave. Throughout this manuscript the term *impedance* refers to the magnitude of the impedance vector, unless otherwise stated. In the relationship between impedance and frequency, the ratio of the impedance at the resonance peak (Z_res_) to the impedance at 0.1 Hz (Z_0_) is called *Q-*value and this parameter is used to highlight the absence (Q < 1.05) or the presence (Q ≥ 1.05) of resonance and its relative strength. The complex representation of the impedance integrates the information of both magnitude and phase. The impedance magnitude corresponds to the length of the vectors connecting the origin of the axes to each point of the graph, while the phase is the angle between each vector and the real axis (see Figure 2D). For resonant cells, the complex representation is characterized by points in both positive and negative regions of the imaginary axes whereas non-resonant neurons display a series of points limited to the negative imaginary region.

### Chemicals and Drugs

All drugs were added to the aCFS medium and bath perfused at the following final concentrations: 10 μM 4-Ethylphenylamino-1,2-dimethyl-6-methylaminopyrimidin chloride (ZD7288, Abcam; HCN channel blocker), 1 μM tetrodotoxin (TTx, Alomone Labs; voltage-dependent Na^+^ channel blocker), 10 μM 2,3-Dioxo-6-nitro-1,2,3,4-tetrahydrobenzo[f]quinoxaline-7-sulfonamide (NBQX, Tocris; AMPA receptors antagonist), 30 μM (RS)-3-(2-Carboxypiperazin-4-yl)-propyl-1-phosphonic acid [(RS)-CPP, Tocris; NMDA receptors antagonist] and 10 μM bicuculline methiodide (Sigma-Aldrich; GABA_A_ receptors antagonist).

### Computational Model

A conductance-based single compartment model reproducing subthreshold resonance was developed using MATLAB. It includes a passive leak current (I_leak_), a hyperpolarization-activated cation current (I_h_), and a persistent (non-inactivating) Na^+^ current (I_NaP_). In the model, these currents were described using the following equations:

(5) $$I_{\text{leak}}=g_{\text{leak}}\times \left(V-E_{\text{leak}}\right)$$

(6) $$I_{\text{h}}=g_{\text{h}}\times f\times \left(V-E_{\text{h}}\right)$$

(7) $$I_{\text{Nap}}=g_{\text{Nap}}\times w\times \left(V-E_{\text{Nap}}\right)$$

with g_leak_, g_h_, and g_NaP_ being the maximal conductances of the corresponding currents and E_leak_, E_h_, and E_NaP_ their reversal potentials.

Moreover, the dynamics of the state variables x_i_ = f were described by the following equation:

(8) $$\frac{{\mathrm{dx}}_{n}}{\mathrm{dt}}=\frac{x_{n\,\infty }\,\left(V\right)-x_{n}}{\tau_{\mathrm{xn}}}$$

where x_n∞_ were the steady-state values of x_n_ and τ_xn_ were the corresponding time constants.

A summary of the reversal potentials and the equations that define the steady-state variables and time constants for the different currents is shown in Table 1. Voltage dependence of state variables and time constants for I_h_ and for I_NaP_ were taken from Spain et al. (1987) and Hodgkin and Huxley (1952), respectively.

**TABLE 1:** Parameters and equations used for calculation of ionic currents in the computational model.

Current	g (ms/cm^2^)	V_rev1_	V_rev2_	State variables	τ (ms)
I_leak_	0.03	−89	−89	−	−
I_h_	0.009	−25	−25	$f_{\infty }=\frac{1}{1+e^{\left(V+70\right)}/7}$	38
I_NaP_	0.23	70.6	74.2	$w_{\infty }=\frac{1}{1+e^{-\left(V+70\right)}/5}$	5

*Summary of conductance values (g), reversal potentials, equations ruling steady-state variables, and time constants (τ) for ionic currents implemented in the model. Voltage dependence of state variables and time constants were taken from Spain et al. (1987) for I_h_ and from Hodgkin and Huxley (1952) for I_NaP_.*

The τ value for I_h_ was divided by the temperature-correcting factor (Magee, 1998):

$$4.5^{\left(T-38\right)/10}$$

where T is the temperature in degree (Celsius).

The model reproduces the stimulus current used in the experiments (I_Zap_). A holding current (I_cmd_) was also added to the model to maintain a holding potential of −70 mV.

The output of the model is the variation of voltage vs. time, which is represented by the following equation:

(9) $$\frac{\mathrm{dV}}{\mathrm{dt}}=\frac{I_{\text{Zap}}-I_{\text{leak}}-I_{\text{h}}-I_{\text{Zap}}+I_{\text{Cmd}}}{C}$$

in which C is the membrane capacitance, fixed at 1 μF/cm^2^.

### Statistics

Collected data are presented as all-point plots together with summary statistics [means ± standard error of the mean (SEM.)] to show them in detail and avoid misinterpretations (Marder and Taylor, 2011; Rathour and Narayanan, 2019). Statistical significance was determined, depending on data, by paired or unpaired two-tailed Student’s *t*-test, One-Way ANOVA test followed by Bonferroni post-hoc, or Kruskal-Wallis test with Dunn’s multiple comparisons test. To assess pairwise relationship in the parameters under investigation we analyzed the scatterplot matrices of them and computed the correspondent Pearson’s correlation coefficients and significance values, as in Mishra and Narayanan (2020).

## Results

### Properties and Firing Patterns of Pyramidal Neurons and GABAergic Interneurons of the Mouse PRC

**FIGURE 1:**
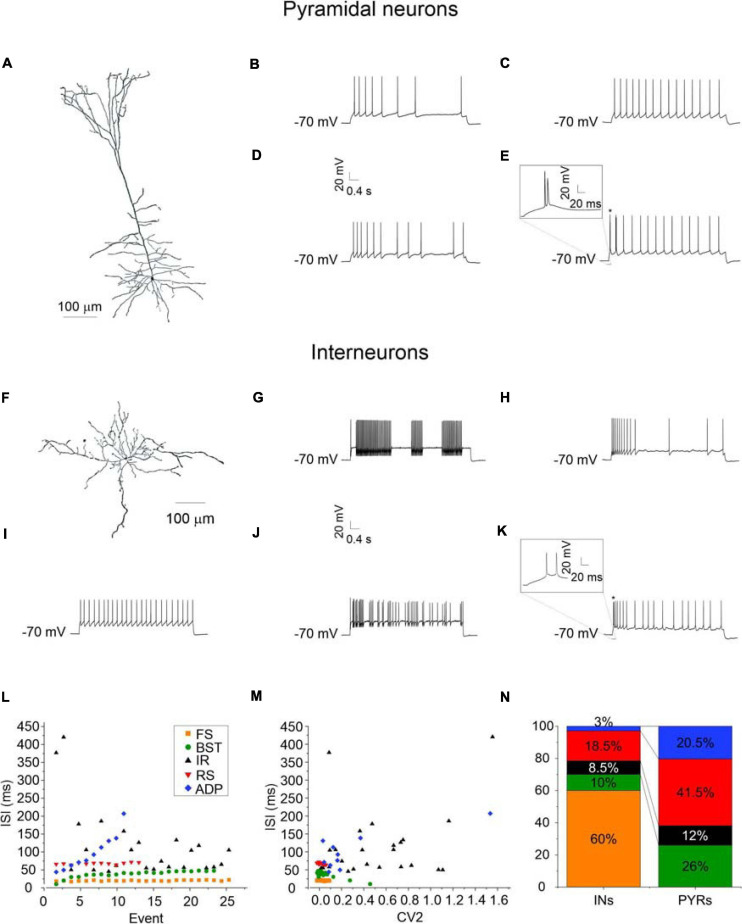
Morphology and firing patterns of pyramidal neurons and GABAergic interneurons of the PRC. **(A)** Imaris reconstruction of a biocytin-labeled pyramidal neuron of the PRC. **(B–E)** Firing traces recorded from representative adapting (ADP) **(B)**, late- and regular-spiking (RS) **(C)**, irregular (IR) **(D)**, and bursting (BST; burst magnification in the inset) **(E)** PRC pyramidal neurons. **(F)** Imaris reconstruction of a biocytin-labeled perirhinal GABAergic interneuron. **(G–K)** Firing traces recorded from representative stuttering fast-spiking (FS) **(G)**, adapting (ADP) **(H)**, late- and regular-spiking (RS) **(I)**, irregular (IR) **(J)**, and bursting (BST; burst magnification in the inset) **(K)** PRC GABAergic interneurons. **(L,M)** Inter-spike-interval (ISI) to event plot **(L)** and inter-spike-interval (ISI) to coefficient of variation (CV2) plot **(M)** showing the behavior of five representative cells, one for each type of firing patter identified in the PRC. **(N)** Percentage of each type of discharge in PRC pyramidal cells (*N* = 128) and GABAergic interneurons (*N* = 70), respectively.

Targeted whole-cell patch-clamp recordings were performed on both deep and superficial layer pyramidal cells and GABAergic interneurons from coronal PRC (areas 35 and 36) brain slices obtained from young GAD67-GFP knock-in mice. In this animal model GABAergic interneurons can be easily identified because they are constitutively labeled with GFP (Tamamaki et al., 2003). In a subset of cells, confirmation of anatomical identity was obtained by biocytin labeling and imaging (Figures 1A,F). From a functional point of view, we found significant differences in passive membrane properties between pyramidal cells and GABAergic interneurons, as expected (Karayannis et al., 2007). Specifically, membrane capacitance (C_m_) was significantly higher in pyramidal neurons than in GABAergic interneurons (*p* < 0.001), while membrane input resistance (R_in_) was significantly higher in GABAergic interneurons as compared with pyramidal neurons (*p* < 0.01) (Table 2). Instead, we found no differences in resting membrane potential (V_r_) (Table 2). Then, we analyzed the firing patterns of the PRC pyramidal neurons and GABAergic interneurons by calculating (i) the distribution of the inter-spike-intervals (ISI) (Figure 1L) and (ii) the coefficient of variation of the discharge (CV2), that measures the intrinsic variability of a spike train (Figure 1M; Holt et al., 1996; Shinomoto et al., 2009; Komendantov et al., 2019; Graf et al., 2020).

**TABLE 2:** Comparison of the principal passive electrophysiological properties of pyramidal neurons and GABAergic interneurons of the PRC.

	Number of cells (N)	C_m_ (pF)	R_in_ (MΩ)	V_r_ (mV)
**Pyramidal neurons**	128	92.5 ± 2.4	181 ± 5	−67.8 ± 0.5
**Interneurons**	70	34.4 ± 1.2 ***	210 ± 10 **	−67.9 ± 0.7

Values of membrane capacitance, membrane input resistance, and membrane resting potential for each cell type are reported as mean ± SEM and were statistically compared (pyramidal neurons vs. GABAergic interneurons) by unpaired Student’s t-test. **p < 0.01; ***p < 0.001.

In this way, we identified 5 different types of discharge (Figures 1B–E,G–K).

(1) Late-spiking regular neurons (RS): At just-suprathreshold, these neurons show a slow ramp depolarization before the onset of their spike trains, with a consequent delay of the first spike. At more sustained depolarizations, they are characterized by a persistent tonic or slightly adapting firing. In line with this, their ISI distribution is linear and almost parallel to the *X*-axis (red dots in Figure 1L). Also, their ISI-CV2 relationship shows a cloud of dots very concentrated and close to each other at a low CV2 (about 0.1) (red dots in Figure 1M).
(2) Stuttering fast-spiking neurons (FS): At just-suprathreshold, these neurons fire trains of high-frequency spikes (30–50 Hz) separated by variable periods of silence. At more sustained depolarizations, they are characterized by a persistent high-frequency (50–100 Hz) tonic firing. Likewise in RS neurons, their ISI distribution is linear and almost parallel to the *X*-axis and their ISI-CV2 relationship shows a cloud of dots very concentrated and close to each other at a low CV2 (about 0.1). However, the FS dots (orange) can be distinguished from the RS dots (red) because they are shifted to lower ISI values (Figures 1L,M).
(3) Adapting neurons (ADP): Adapting neurons typically begin their spike trains at a short latency following onset of a depolarizing current step and accommodate strongly. Due to adaptation, their ISI distribution is linear, but with a higher angular coefficient than RS and FS neurons (blue dots in Figure 1L). Also, their ISI-CV2 relationship shows a quite dispersed cloud of dots (blue dots in Figure 1M) with a higher mean CV2 (about 0.4).
(4) Bursting neurons (BST): Bursting neurons are characterized by spikes that occur in a stereotyped pattern consisting into a cluster of 2–3 action potentials riding on a slow depolarizing wave and followed by a strong slow afterhyperpolarization. After the burst, their firing generally becomes regular. Therefore, their ISI distribution is not linear but starts with shorter ISIs (green dots in Figure 1L) and their ISI-CV2 relationship consists in a rather compact cloud of dots (corresponding to the regular firing) accompanied by two or three more dispersed dots (corresponding to the burst) (green dots in Figure 1M).
(5) Irregular neurons (IR): Irregular neurons show a random and unpredictable firing pattern. Their ISI distribution is dispersed and not linear (black dots in Figure 1L) and also their ISI-CV2 relationship consists in a dispersed cloud of dots (black dots in Figure 1M). They have a mean CV2 similar to that of adapting neurons (about 0.4), but their non-linear ISI distribution uniquely characterizes them.

This classification is similar to what is currently described in literature (Faulkner and Brown, 1999; Beggs et al., 2000; McGann et al., 2001). Accordingly, we found that 41.5% of pyramidal neurons were RS, 20.5% ADP, 26% BST, and 12% IR. The same classification indicates that 60% of GABAergic interneurons were FS, 18.5% RS, 3% ADP, 10% BST, and 8.5% IR (Figure 1N).

### Resonant and Non-resonant Behavior in Glutamatergic Pyramidal Neurons and GABAergic Interneurons of the PRC

**FIGURE 2:**
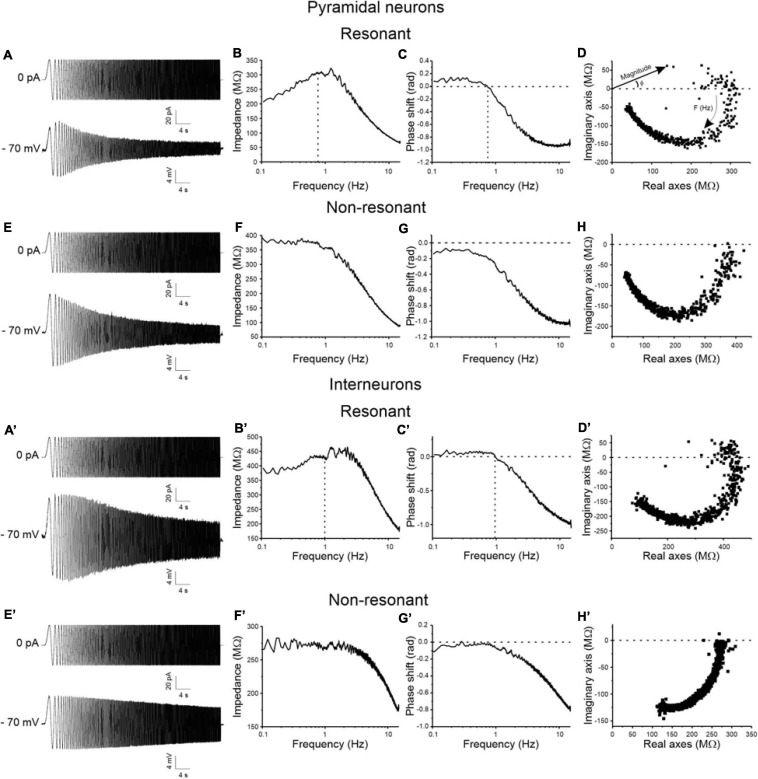
Resonant vs. non-resonant behavior of pyramidal neurons **(A–H)** and GABAergic interneurons **(A′–H′)** of the PRC. **(A)** Representative voltage response of a resonant pyramidal neuron (lower trace) to a ZAP input of linearly increasing frequency from 0 to 15 Hz (upper trace) at membrane potential of –70 mV. **(B)** Impedance magnitude vs. frequency relationship of the same cell as in **(A)**. Dashed line in correspondence to the peak in the relationship indicates the F_res_ of the cell. **(C)** Impedance phase vs. frequency relationship of the same cell as in **(A)**. The resonant behavior of the cell determines a positive phase shift at the low frequencies, reflecting anticipation of the voltage wave relative to the current wave. Hence, F_res_ corresponds to the value of frequency at which phase shift is 0 rad (dashed lines). **(D)** Complex representation of impedance of the same neuron shown in **(A–C)**. Impedance vectors are represented as points in the complex plane: the length of the vectors connecting the origin of the axes to each point of the graph corresponds to the impedance magnitude and the angle between each vector and the real axis corresponds to the impedance phase. Note that for resonant cells the complex representation is characterized by points in both positive and negative regions of the imaginary axis. **(E–H)** Same as **(A–D)**, for a representative non-resonant pyramidal neuron. The non-resonant behavior of the cell is evidenced by the absence of the peak in the impedance-to-frequency relationship **(F)**, the negative phase shift at all investigated frequencies **(G)**, and the points limited to the negative imaginary region of the impedance locus diagram **(H)**. **(A′–H′)** Same as **(A–H)** for a representative resonant **(A′–D′)** and a representative non-resonant **(E′–H′)** GABAergic interneuron.

**FIGURE 3:**
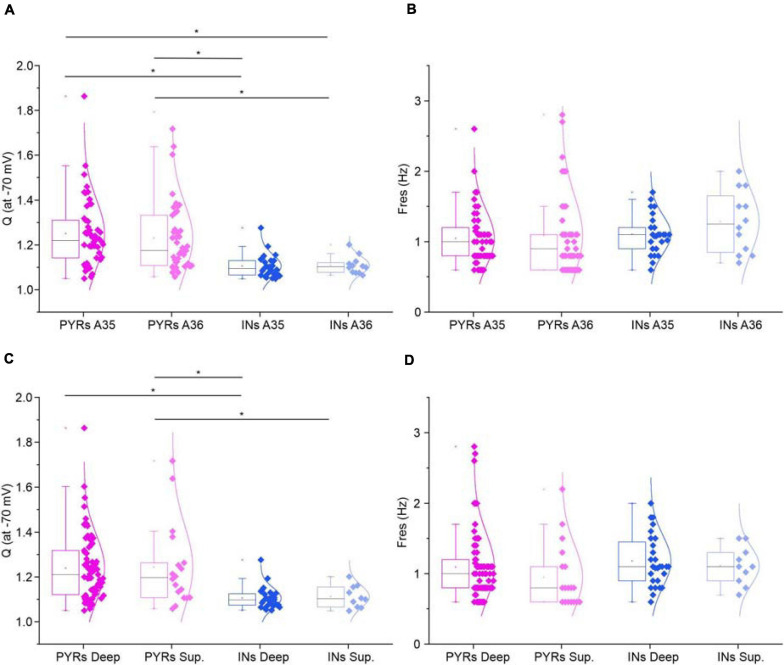
Resonance in the different areas and layers of the PRC. **(A,B)** All-points half-violin plots flanked by summary box plots for Q **(A)** and F_res_ **(B)** at –70 mV for resonant pyramidal neurons and GABAergic interneurons of Area35 vs. Area 36. **(C,D)** Same as **(A–D)**, for superficial vs. deep layers. Data were compared by One-Way ANOVA followed by Bonferroni *post hoc*. *p < 0.05.

A regular 50sec-long ZAP current input with linearly increasing frequency from 0 to 15 Hz was applied to test for resonant behavior of pyramidal neurons (Figures 2A,E) and GABAergic interneurons (Figures 2A′,E′) at a membrane potential of −70 mV. Resonance appears as a peak in the voltage response at a specific frequency (F_res_) (Figures 2A,A′), that is absent in non-resonant cells (Figures 2E,E′). As a consequence, resonant cells show a peak in the impedance-to-frequency relationship at F_res_ (corresponding to the dashed vertical line in Figures 2B,B′), whereas a clear peak is not detectable in non-resonant cells (Figures 2F,F′). Accordingly, the phase shift-to-frequency relationship and the complex representation of the impedance differentiates between resonant (Figures 2C,D,C′,D′) and non-resonant (Figures 2G,H,G′,H′) neurons, through clustering of positive values in resonant neurons. The percentage of the resonant pyramidal neurons and GABAergic interneurons measured in the superficial and deep layers of areas 35 (A35) and 36 (A36) of the PRC is shown in Table 3. Overall, the majority of perirhinal pyramidal neurons (77%) and GABAergic interneurons (54%) were resonant and were equally distributed throughout the PRC, without a clear prevalence in a specific area or layer (Table 3), suggesting that resonance could be very important for the oscillatory synchronization and integration of the neuronal activity in this region. Also, the resonance strength (Q_–__70_) and the frequency of resonance (F_res_) were similar in pyramidal neurons of A35 vs. A36 and of superficial vs. deep layers (Figures 3A–D). Comparable results were obtained also in GABAergic interneurons (Figures 3A–D). However, we found that the Q_–__70_ was significantly different between pyramidal neurons and GABAergic interneurons regardless of their location (Figures 3A,C).

**TABLE 3:** Distribution of the resonant pyramidal neurons and GABAergic interneurons in the superficial and deep layers of the areas 35 (A35) and 36 (A36) of the PRC.

	A35		A36		Total		Total	
				
	Superficial layers	Deep layers	Superficial layers	Deep layers	A35	A36	Superficial layers	Deep layers
**Pyramidal neurons**	10/15 (67%)	44/55 (80%)	9/17 (53%)	36/41 (88%)	54/70 (77%)	45/58 (78%)	19/32 (59%)	80/96 (83%)
**Interneurons**	6/12 (50%)	20/40 (50%)	4/5 (80%)	8/13 (62%)	26/52 (50%)	12/19 (63%)	10/17 (59%)	28/53 (53%)

*Number of resonant cells vs. total number of recorded cells and corresponding percentages. Resonant neurons are numerous and equally distributed throughout the PRC, without a clear prevalence in a specific area or layer.*

In a subset of cells, experiments were repeated using “inverted” (*N* = 13) and “negative” (*N* = 12) ZAP protocols without any changes in the reported resonant properties of the cells (data not shown). This demonstrates that the resonant peak depends on the frequency of stimulation and not on the timing or orientation of the ZAP input. The ZAP input was also applied before and following perfusion of the synaptic blockers NBQX (10μM), (RS)-CPP (30 μM), and bicuculline methiodide (10 μM), without any changes in resonant properties demonstrating that membrane resonance is an intrinsic property of the neurons and is not driven by synaptic input (*N* = 5; not shown).

### Correlations in Firing Properties, Passive Properties, and Resonant/Non-resonant Behavior of the PRC Neurons

**FIGURE 4:**
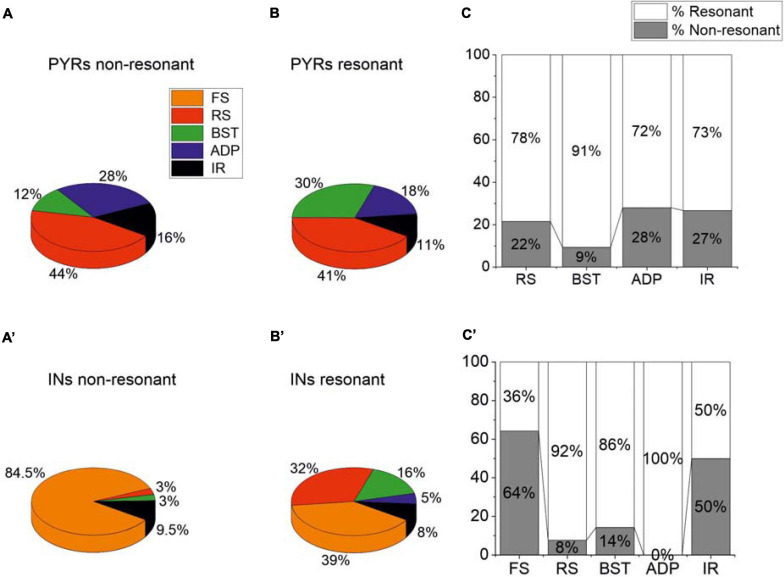
Relationships between firing patterns and resonant or non-resonant behavior of the PRC neurons. **(A,B)** Percentage of specific firing patterns for resonant **(A)** and non-resonant **(B)** PRC pyramidal neurons. **(C)** Percentage of resonant vs. non-resonant cells classified for a specific firing pattern of pyramidal neurons. **(A′–C′)** Same as **(A–C)** for GABAergic interneurons.

As already stated, PRC pyramidal neurons and GABAergic interneurons showed different firing patterns in response to the injection of suprathreshold current steps. Therefore, we tried to identify possible correlations between the firing pattern and the resonant/non-resonant behavior of these cells. To this purpose, we computed the percentage of specific firing patterns for non-resonant and resonant pyramidal (Figures 4A,B) and GABAergic neurons (Figures 4A′,B′). Furthermore, we computed the percentage of resonant vs. non-resonant cells classified for a specific firing pattern of pyramidal (Figure 4C) and GABAergic neurons (Figure 4C′). In pyramidal neurons, we found a prevalence of resonant neurons regardless of the firing pattern (Figure 4C). In addition, the percentage of the different types of firing pattern was quite similar in non-resonant (Figure 4A) and resonant (Figure 4B) cells. In contrast, in GABAergic interneurons we found (i) a large prevalence of resonant cells in RS, BST, and ADP neurons, (ii) an equal percentage of resonant vs. non-resonant cells in IR neurons, and iii) a prevalence of non-resonant cells in FS neurons (Figure 4C′). Resonant interneurons showed all the types of firing patterns, although in different percentages (Figure 4B′). On the contrary, a high percentage (84.5%) of non-resonant GABAergic interneurons were FS (Figure 4A′).

**FIGURE 5:**
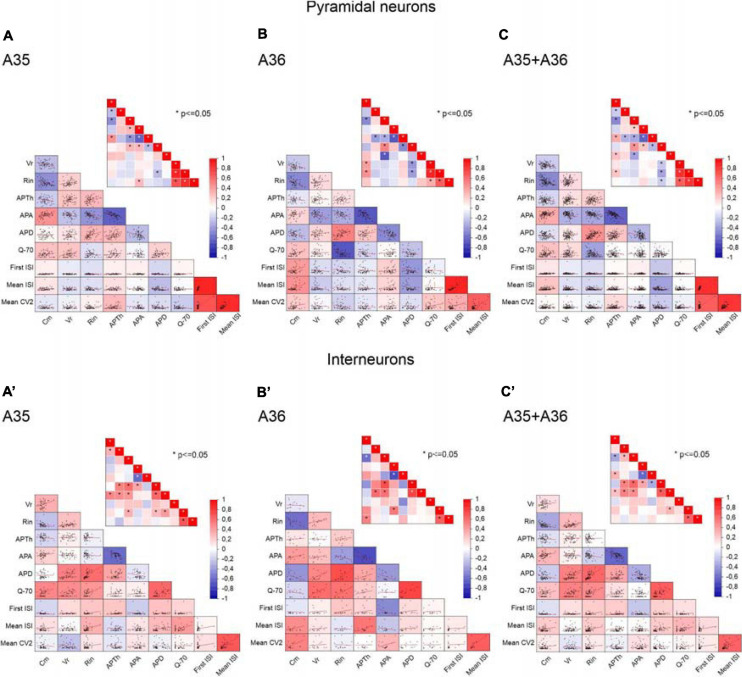
Correlations between passive properties, firing properties, and resonance in the neurons of the different PRC areas. **(A–C)** Pairwise scatterplot matrices of C_m_ (membrane capacitance), V_r_ (membrane resting potential), R_in_ (input resistance), AP_Th_ (spike Threshold), AP_A_ (spike Amplitude), AP_D_ (spike Duration), Q_–__70_, First ISI, Mean ISI, Mean CV2 of pyramidal neurons recorded from A35 **(A)**, A36 **(B)**, and both A35+A36 **(C)**. These scatterplot matrices are overlaid on the corresponding color-coded correlation matrices and the insets in each panel represent the significance value associated with each scatterplot, computed as Pearson’s correlation coefficients. **(A′–C′)** Same as **(A–C)** for GABAergic interneurons. *p ≤ 0.05.

**FIGURE 6:**
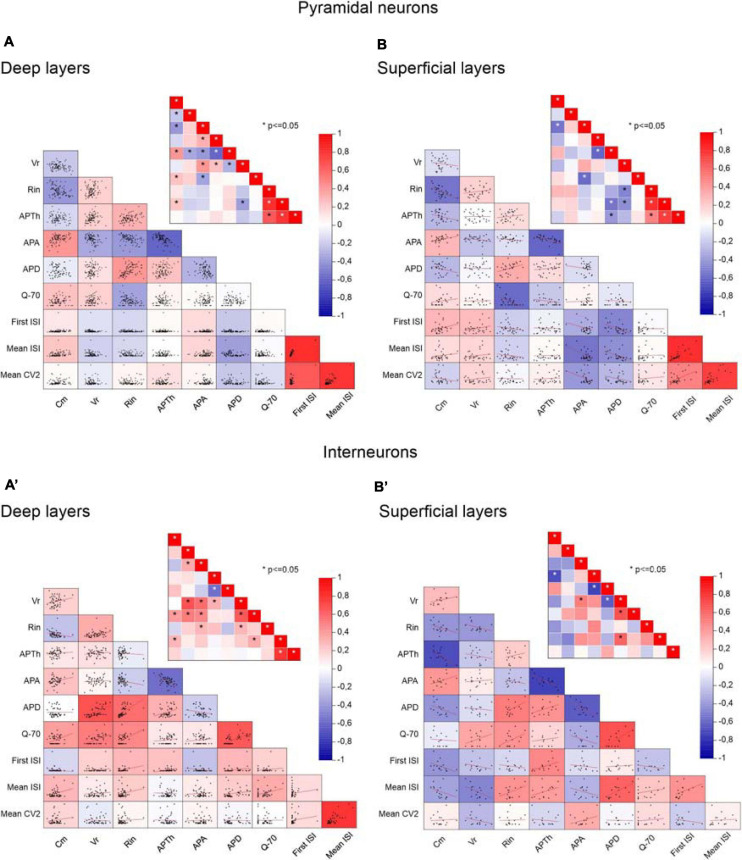
Correlations between passive properties, firing properties, and resonance in the neurons of the different PRC layers. **(A,B)** Pairwise scatterplot matrices of C_m_ (membrane capacitance), V_r_ (membrane resting potential), R_in_ (input resistance), AP_Th_ (spike Threshold), AP_A_ (spike Amplitude), AP_D_ (spike Duration), Q_–__70_, First ISI, Mean ISI, Mean CV2 of pyramidal neurons recorded from the deep **(A)** and the superficial **(B)** layers. These scatterplot matrices are overlaid on the corresponding color-coded correlation matrices and the insets in each panel represent the significance value associated with each scatterplot, computed as Pearson’s correlation coefficients. **(A′,B′)** Same as **(A,B)** for GABAergic interneurons. *p ≤ 0.05.

To clarify the interdependencies between passive properties, firing properties, and resonance in the neurons of the different PRC areas and layers, we plotted pairwise scatterplots of C_m_ (membrane capacitance), R_in_ (input resistance), V_r_ (membrane resting potential), AP_Th_ (spike Threshold), AP_A_ (spike Amplitude), AP_D_ (spike Duration), First ISI, Mean ISI, Mean CV2, Q_–__70_ recorded from pyramidal and GABAergic neurons of A35 and A36 (Figure 5) and of superficial and deep layers (Figure 6) of the PRC. Then we computed Pearson’s correlation coefficients for each of them. By examining separately pyramidal neurons or GABAergic interneurons, we found that the correlation matrices were quite similar among the different areas (Figure 5) and layers (Figure 6). As expected, we found significant correlations in pairwise comparisons of passive properties (for example inverse correlation between C_m_ and R_in_) and of firing properties (for example inverse correlation between AP_Th_ and AP_A_; direct correlation between Mean ISI and Mean CV2).

Focusing on Q_–__70_, in pyramidal neurons we found that this parameter was significantly correlated only with R_in_ (inverse correlation) (Figures 5A–C, 6A,B). Furthermore, by directly comparing R_in_ in resonant vs. non-resonant pyramidal neurons, we found a significantly lower R_in_ in resonant cells (Table 4). We also found a significantly more depolarized V_r_ in resonant pyramidal neurons (Table 4). Overall, we assume these results to be due to the presence of a putative cationic current expressed only in resonant cells and active at their V_r_ (−70 mV).

**TABLE 4:** Comparison of the principal passive electrophysiological properties of resonant vs. non-resonant neurons of the PRC.

	Number of cells (N)		C_m_ (pF)		R_in_ (MΩ)		V_r_ (mV)	
				
	Resonant	Non-resonant	Resonant	Non-resonant	Resonant	Non-resonant	Resonant	Non-resonant
**Pyramidal neurons**	99	29	94.5 ± 2.5	85.8 ± 6.3	175 ± 5 *	202 ± 15	−67.1 ± 0.5 **	−70.3 ± 1.0
**Interneurons**	38	32	35.7 ± 1.9	32.9 ± 1.3	237 ± 15 **	177 ± 9	−65.9 ± 0.8 ***	−70.3 ± 0.9

Values of membrane capacitance, membrane input resistance, and membrane resting potential for resonant vs. non-resonant neurons are reported as mean ± SEM and were statistically compared by unpaired Student’s t-test. *p < 0.05; **p < 0.01; ***p < 0.001.

In GABAergic interneurons we found significant correlations between Q_–__70_ and many other passive (direct correlations with C_m_, V_r_, and R_in_) and firing (direct correlations with Mean ISI and AP_D_) parameters (Figures 5A′–C′, 6A′,B′). This suggested an effective correlation between the firing patterns and the resonant/non-resonant behavior of the GABAergic interneurons, as already indicated by the percentage distributions in Figure 4. In fact, non-resonant GABAergic interneurons were mainly FS cells characterized, compared to the other subclasses of interneurons, by higher firing rate (lower Mean ISI), lower AP_D_, lower C_m_, and lower R_in_ (Ma et al., 2006). Interestingly, the direct comparison of R_in_ in resonant vs. non-resonant GABAergic interneurons reinforced this hypothesis since we found a significantly lower R_in_ in non-resonant cells (Table 4). As in pyramidal neurons, we also found a significantly more depolarized V_r_ in resonant GABAergic neurons (Table 4), likely due to both the higher R_in_ and the expression of a resonant-specific cationic current.

### Resonance Properties at Different Membrane Potentials

**FIGURE 7:**
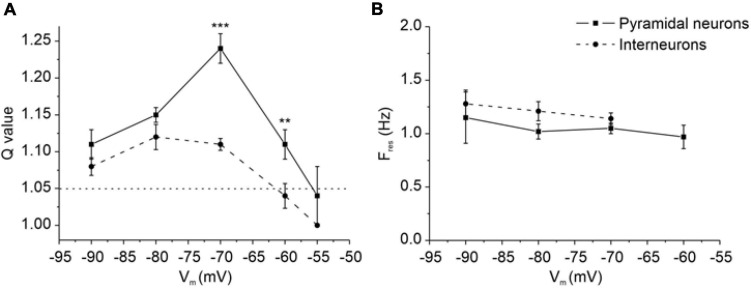
Resonance properties of PRC pyramidal neurons and GABAergic interneurons at different membrane potentials. **(A)** Average Q values at different membrane potentials for pyramidal neurons (solid line) and GABAergic interneurons (dashed line). *Q*-value is defined as the ratio of impedance magnitude at F_res_ and 0.1 Hz and is used to highlight the strength of the resonance. Dotted line at *Q* = 1.05 discriminates between absence (*Q*-value < 1.05) and presence (*Q*-value ≥ 1.05) of resonance. On average, pyramidal neurons show a significantly higher *Q*-value at –60 and –70 mV (*N* = 35 and *N* = 99, respectively) compared to GABAergic interneurons recorded at the same membrane potentials (*N* = 15 and *N* = 37, respectively) (**p < 0.01; ***p < 0.001; unpaired Student’s *t*-test). Moreover, among pyramidal neurons the mean *Q*-value was greater at –70 mV (*N* = 99) compared to the most of other tested potential [–55 mV (*p* < 0.01, *N* = 5), –60 mV (*p* < 0.001, *N* = 35), –80 mV (*p* < 0.001, *N* = 40); Kruskal-Wallis followed by Dunn’s multiple comparisons test]. Among interneurons the mean *Q*-value was lower at –60 mV (*N* = 15) compared to –70 mV (*p* < 0.001, *N* = 37) and –80 mV (*p* < 0.01, *N* = 12) (Kruskal-Wallis followed by Dunn’s multiple comparisons test). **(B)** Average F_res_ at different membrane potentials for pyramidal neurons (solid line) and GABAergic interneurons (dashed line). The mean values of F_res_ are similar between pyramidal neurons [–60 mV (*N* = 21), –70 mV (*N* = 99), –80 mV (*N* = 38) and –90 mV (*N* = 8)] and GABAergic interneurons [–70 mV (*N* = 37), –80 mV (*N* = 12), –90 mV (*N* = 9)] at all tested potentials (unpaired Student’s *t*-test).

In resonant neurons, the voltage-dependence of Q and F_res_ was tested by applying the ZAP protocol at different membrane potentials, ranging from −55 to −90 mV. In pyramidal neurons (solid line), resonance (*Q*-values > 1.05) began at potential more negative than −55 mV and was preserved at more hyperpolarized potentials (Figure 7A). Resonance strength was maximal at −70 mV, as emphasized by the relative increase in the *Q*-value at this potential (Q = 1.24 ± 0.02, Figure 7A).

In contrast, GABAergic interneurons (dashed line) start to show resonance at potentials more negative than −60 mV and did not show a clear increase in the *Q*-value at any specific potential (Figure 7A). It is noteworthy that *Q*-values were significantly greater in pyramidal neurons than in GABAergic interneurons at both −60 mV (*p* < 0.01) and −70 mV (*p* < 0.001), perhaps suggesting that pyramidal neurons express an amplifying conductance that increases the *Q*-values which might be not present in interneurons.

F_res_ was similar in the two cell types and nearly constant (1–1.5 Hz) in a wide range of membrane potentials (Figure 7B).

### Effects of the Block of HCN Channels on Resonant Behavior of the Perirhinal Neurons

As stated above, we found a more depolarized membrane resting potential in resonant compared to non-resonant neurons, suggesting the presence of a putative cationic conductance expressed only in resonant cells and active at V_r_ (−70 mV). Moreover, resonance behavior was preserved at hyperpolarized potentials, but disappeared at depolarized potentials. Based on this evidence and on literature (Hutcheon et al., 1996; Hu et al., 2002; Nolan et al., 2007; Biel et al., 2009; Ehrlich et al., 2012; Boehlen et al., 2013; Vera et al., 2014), we hypothesized the involvement of an I_h_ current in the resonance of PRC neurons. I_h_ is indeed a cationic subthreshold current showing biophysical properties that could cause low-frequency resonance at hyperpolarized potentials.

**FIGURE 8:**
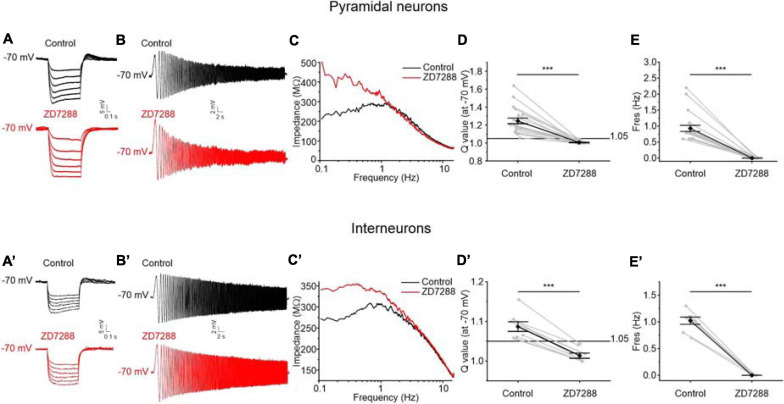
Effects of the block of HCN channels on resonant behavior of pyramidal neurons **(A–D)** and GABAergic interneurons **(A′–D′)** of the PRC. **(A)** Representative voltage traces evoked by applying hyperpolarizing current steps to a resonant pyramidal neuron. A prominent “*sag”* appears in the voltage responses (upper traces in black) and is abolished after administration of the HCN blocker ZD7288 10 μM (lower traces in red). **(B)** Voltage responses of a representative resonant pyramidal neuron to the standard 0–15 Hz ZAP protocol at –70 mV before (upper trace in black) and after (lower trace in red) the perfusion of ZD7288 10 μM. **(C)** Impedance-to-frequency relationship of the same cell as in **(B)**, before (black line) and after (red line) ZD7288 perfusion. **(D,E)** Scatter plots comparing the *Q*-values **(C)** and F_res_ values **(D)** derived from resonant pyramidal neurons (*N* = 21) before (Control) and after (ZD7288) blocking the HCN channels (gray dots and lines). Mean ± SEM. *Q*-values are reported on the same plot in black. In ZD7288 condition, the resonant behavior is completely abolished. **(A′–D′)** Same as **(A–D)** for GABAergic interneurons (*N* = 8): also in GABAergic interneurons ZD7288 administration completely abolishes “*sag*” **(A′)** and resonant behavior **(B′–D′)**. Data in **(D,E,D′,E′)** were compared by paired Student’s *t*-test. ***p < 0.001.

Consistently with the hypothesis that I_h_ is implicated in PRC resonance, we found a prominent “sag” in the voltage response to hyperpolarizing current steps, likely due to I_h_ activation, in all resonant perirhinal neurons (Figures 8A,A′), but not in non-resonant neurons. The “sag” was completely abolished by the administration of ZD7288 (10 μM), a specific blocker of HCN (hyperpolarization-activated cyclic nucleotide–gated) channels whose activation generate the I_h_ current in neurons (Figures 8A,A′).

Following blockage of I_h_ by ZD7288, the expression of resonant behavior was completely blocked (Figures 8B–E,B′–E′). The effect of the inhibition of I_h_ on the ZAP response is shown in the example traces reported in Figures 8B,B′. Following ZD7288 application the peak in the impedance-to-frequency relationship was abolished (Figures 8C,C′) and the *Q*-value decreased below the resonance threshold (Figures 8D,D′) for both resonant pyramidal cells and GABAergic interneurons, confirming a key role of I_h_ in generating resonance in PRC.

### Role of Persistent Sodium Current in Amplifying the Resonant Behavior of Perirhinal Pyramidal Neurons

**FIGURE 9:**
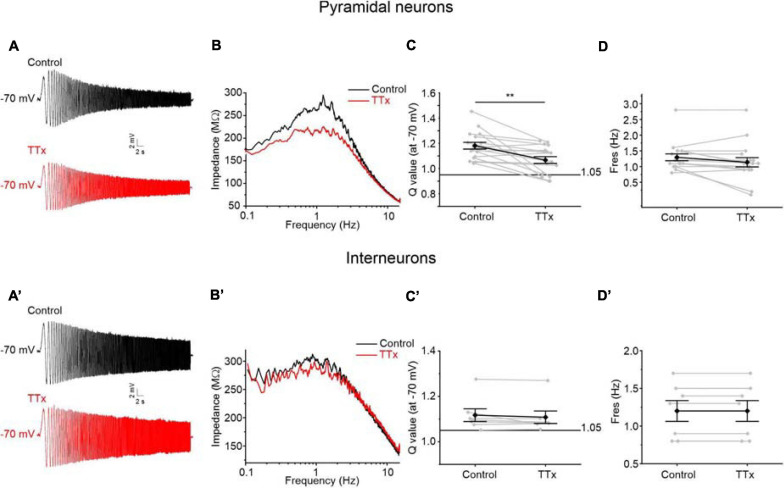
Effects of the block of TTx-sensitive sodium channels on resonant behavior of pyramidal neurons **(A–D)** and GABAergic interneurons **(A′–D′)** of the PRC. **(A)** Voltage responses of a representative resonant pyramidal neuron to the standard 0–15 Hz ZAP protocol at –70 mV before (upper trace in black) and after (lower trace in red) the perfusion of TTx 1 μM. **(B)** Impedance-to-frequency relationship of the same cell as in **(B)**, before (black line) and after (red line) TTx perfusion. **(C,D)** Scatter plots comparing the *Q*-values **(C)** and F_res_ values **(D)** derived from resonant pyramidal neurons (*N* = 17) before (Control) and after (TTx) blocking the TTx-sensitive Sodium channels (gray dots and lines). Mean ± SEM. *Q*-values are reported on the same plot in black. In TTx condition, the *Q*-value is significantly reduced and then the resonance strength is attenuated **(C)**. The F_res_ is unaltered by TTx administration **(D)**. **(A′–D′)** Same as **(A–D)** for GABAergic interneurons (*N* = 7). In GABAergic interneurons TTx perfusion has no effect either on the *Q*-value **(C′)** or on the F_res_ **(D′)**. Data in **(C,D,C′,D′)** were compared by paired Student’s *t*-test. **p < 0.01.

Given that the *Q*-value was significantly higher in resonant pyramidal neurons when compared to resonant interneurons at −60 and −70 mV (Figure 7A), we hypothesized that these neurons selectively express an amplifying current that is widely described in several classes of resonant cells (Traub and Miles, 1991; Gutfreund et al., 1995; Hutcheon et al., 1996; Hutcheon and Yarom, 2000; Hu et al., 2002; Vera et al., 2014; Matsumoto-Makidono et al., 2016). Since the persistent sodium current (I_NaP_) is a typical amplifying current that has been already reported to enhance the resonant behavior at membrane potentials near V_r_ (Hutcheon et al., 1996; Vera et al., 2014), we tested the involvement of I_NaP_ on the amplification of the membrane resonance in PRC pyramidal neurons. To this purpose, we applied TTx (1μM) during the administration of the ZAP protocol. We found that TTx attenuates the resonance strength measured at −70 mV in pyramidal neurons (Figures 9A,B) but not in GABAergic interneurons (Figures 9A′,B′). Accordingly, the *Q*-value was significantly reduced from 1.3 to 1.2 in pyramidal cells (*p* < 0.01) (Figure 9C), whereas it remained unchanged in GABAergic interneurons (Figure 9C′). As expected, TTx did not alter the values of F_res_ in both cell type (Figures 9D,D′).

These data confirm that the persistent sodium current has an amplifying effect on the resonant behavior specifically in the pyramidal neurons of the PRC. TTx blocks also the transient Na^+^ current but we excluded the involvement of this current in resonance amplification because of its fast kinetics and its more depolarized activation threshold.

### Effect of Temperature on the Resonance Properties of Perirhinal Neurons

**FIGURE 10:**
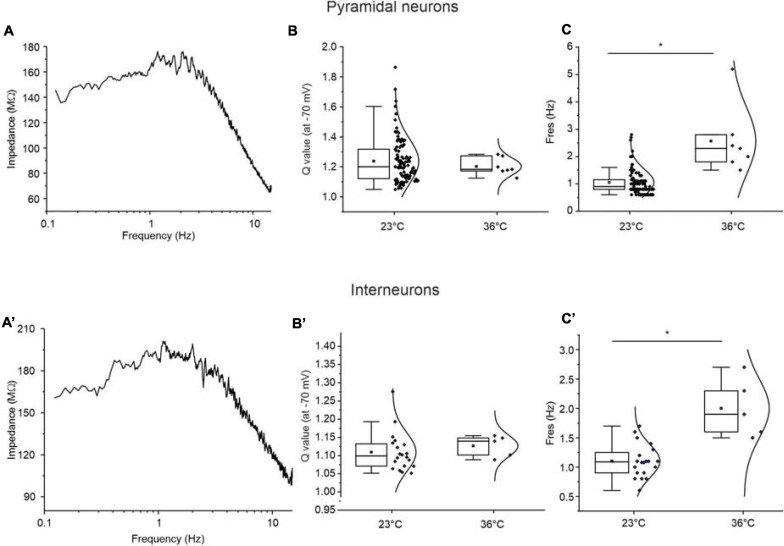
Effect of temperature on the resonance properties of pyramidal neurons **(A–C)** and GABAergic interneurons **(A′–C′)** of the PRC. **(A)** Impedance-to-frequency relationship obtained from a representative resonant pyramidal neuron at a temperature of 36°C by applying the standard 0–15 Hz ZAP protocol at –70 mV. **(B,C)** All-points half-violin plots flanked by summary box plots comparing the *Q*-values **(B)** and F_res_ values **(C)** derived from resonant pyramidal neurons at room temperature (23°C) and at physiological temperature (36°C). The increase of temperature in the 7 pyramidal neurons tested at 36°C has no influence on the *Q*-value **(B)**, while determines a significant increase of F_res_ **(C)**. **(A′–C′)** Same as **(A–C)** for resonant GABAergic interneurons (*N* = 5). Note that also in this cell type, the temperature increase does not alter the *Q*-value **(B′)**, while determines a significant increase of the F_res_ **(C′)**. Data in **(B,C,B′C′)** were compared by unpaired Student’s *t*-test. *p < 0.05.

It has been shown that the resonance properties of the neurons can be affected by temperature (Hu et al., 2002; Yan et al., 2012; Vera et al., 2014). To verify whether the resonance properties of perirhinal neurons are also influenced by temperature, we performed a set of experiments at 36°C. Figures 10A,A′ show representative impedance profiles derived at 36°C from a resonant pyramidal neuron and a resonant GABAergic interneuron, respectively. Data were obtained by applying the standard 0–15 Hz ZAP protocol at −70 mV. The analysis revealed that the *Q*-value does not change by increasing the temperature from 23 to 36° C, either in pyramidal neurons or GABAergic interneurons (Figures 10B,B′). On the contrary, resonant frequency significantly increased from 1.17 ± 0.04 to 2.53 ± 0.49 Hz in pyramidal neurons (*p* < 0.05) and from 1.13 ± 0.04 to 2.00 ± 0.22 Hz in GABAergic interneurons (*p* < 0.05) (Figures 10C,C′).

### Computational Model

The resonant behavior of the perirhinal pyramidal neurons and GABAergic interneurons was reproduced by a computational model in MATLAB (see section “Materials and Methods” for details) by applying the same sinusoidal ZAP input current used in the electrophysiological experiments (Origin 6.0).

**FIGURE 11:**
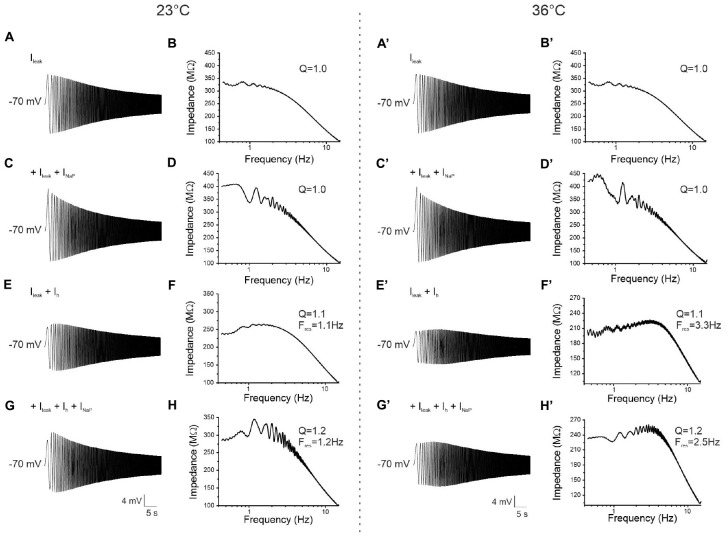
Computational MATLAB model reflecting experimental data and simulating resonance. **(A)** Voltage response to the 0–15 Hz ZAP input at –70 mV and 23°C simulated by a model including only the leakage current (I_leak_). **(B)** Impedance-to-frequency relationship obtained from simulation in **(A)**. The behavior strongly resembles that of an experimentally recorded non-resonant neuron. **(C,D)** Same as **(A,B)**, but with the insertion of the persistent sodium current (I_NaP_) in the model. The voltage response **(C)** and the corresponding impedance-to-frequency relationship **(D)** still resemble that of a non-resonant neuron. **(E,F)** Same as **(A,B)**, but with the insertion of a HCN channels-mediated current (I_h_) in the model. The voltage response **(E)** and the corresponding impedance-to-frequency relationship **(F)** show that in these conditions the model is able to simulate resonant behavior. **(G,H)** Same as **(A,B)** but with the insertion of both I_h_ and I_NaP_ in the model. The voltage response **(G)** and the corresponding impedance-to-frequency relationship **(H)** show that this model not only resonates, thanks to I_h_, but it also displays a resonance amplified by I_NaP_. **(A′–H′)** Same as **(A–H)**, but after setting the value of temperature at 36°C instead of 23°C in the model. Note that in the resonant model at the higher temperature **(E′–H′)** the *Q*-value remains constant while the F_res_ increases, as experimentally found. Model parameters are shown in Table 1.

At first, the model was tested with a ZAP protocol at −70 mV in its basal conditions where only the leakage current (I_leak_) was expressed (Figure 11A). The corresponding impedance profile (Figure 11B) strongly resembled the pattern of a non-resonant neuron acting as a low-pass filter. By adding the persistent sodium current (I_NaP_), the voltage response and the corresponding impedance profile still showed a lack of a resonant behavior (Figures 11C,D) indicating that I_NaP_ alone is not sufficient to generate resonance. A resonant response was obtained by simulating the expression of the h-current (I_h_), as indicated by the appearance of a peak in the impedance profile (*Q* = 1.1) (Figures 11E,F). The F_res_ calculated from the impedance profile obtained in the model overlapped that measured during patch-clamp recording experiments. In a model simulating the presence of I_leak_, I_h_, and I_NaP_ an amplification of the resonance was observed (Figures 11G,H), as demonstrated by an increase of the *Q*-value (*Q* = 1.2) compared to the previous condition.

To examine whether the effect of the temperature on the resonant frequency was also reproducible in the model, the value of the simulated temperature (T) was increased up to 36°C (Figures 11A′–H′). As expected, F_res_ shifted toward higher values in the model simulating the presence of both I_leak_ and I_h_ (Figures 7E′,F′) and in the model simulating the contribution of all three currents I_leak_ + I_h_+ I_NaP_ (Figures 7G′,H′).

In summary, by implementing a computational model with parameters obtained with patch-clamp experiments we were able to reproduce the resonant behavior observed in both pyramidal neurons and GABAergic interneurons of the PRC.

## Discussion and Conclusion

This paper describes for the first time the membrane resonance properties of pyramidal cells and GABAergic interneurons throughout the different layers of the mouse perirhinal cortex. These findings represent a missing piece of a comprehensive puzzle designed to define the functional contribution of the parahippocampal cortical regions in the processing of information directed to and propagated from the hippocampus. In particular, by applying a 0–15 Hz ZAP protocol, we demonstrated that a substantial subpopulation (over 75% of pyramidal cells and over 50% of GABAergic interneurons) of the PRC neurons showed membrane resonance in a frequency (F_res_) ranging from 1 to 2.5 Hz. By using “inverted” and “negative” ZAP protocols, as well as by repeating the experiments before and following perfusion of synaptic blockers NBQX, (RS)-CPP, and bicuculline methiodide, we demonstrated that resonant behavior and peak are intrinsic properties of the PRC cells, not influenced by the time and the orientation of the ZAP input and not driven by synaptic input. It is worth noticing that bicuculline methiodide blocks also SK-channels (Johnson and Seutin, 1997). This is a potential caveat that needs to be considered, since SK-current could possibly be implicated in the generation of resonance (Xue et al., 2012). However, we did not observe changes in resonant properties following the perfusion of the synaptic blockers (including bicuculline methiodide). Then, we believe that SK-current is not implicated in the generation of the resonant response in the PRC cells and the results of the subset of experiments involving the administration of synaptic blockers are reliable.

We did not observed differences in the distribution of the pyramidal and GABAergic resonant neurons throughout the different areas (A35 and A36) and layers (deep and superficial) of the PRC. Furthermore, the resonance strength and the frequency of resonance seemed to be independent from the localization of the cells. The Pearson’s correlation matrices of C_m_ (membrane capacitance), R_in_ (input resistance), V_r_ (membrane resting potential), AP_Th_ (spike Threshold), AP_A_ (spike Amplitude), AP_D_ (spike Duration), First ISI, Mean ISI, Mean CV2, and Q_–__70_ were also similar when computed in different PRC areas and layers.

On the other hand, by analyzing possible correlations between the firing pattern and the resonant/non-resonant behavior of the PRC cells, we found interesting differences between pyramidal neurons and GABAergic interneurons. In pyramidal neurons the percentage of the different types of firing pattern (RS, ADP, BST, IR) was quite similar in non-resonant and resonant cells, with a majority of resonant cells regardless of the firing pattern. In pyramidal neurons, the Q_–__70_ was inversely correlated with R_in_. R_in_ was also significantly lower in cells that showed a resonant behavior and expressed the I_h_ at V_r_. No others significant correlations emerged. In contrast, in GABAergic interneurons Q_–__70_ significantly correlated with many other passive (direct correlations with C_m_, V_r_, and R_in_) and firing (direct correlations with Mean ISI and AP_D_) parameters. The high prevalence of FS cells—characterized by higher firing rate (lower Mean ISI), lower AP_D_, lower C_m_, and lower R_in_ (Ma et al., 2006)—in non-resonant GABAergic interneurons could support these correlations. A significantly lower R_in_ in non-resonant compared to resonant interneurons reinforced this hypothesis. Overall, these data suggested an effective correlation between the firing pattern and the resonant/non-resonant behavior in the GABAergic interneurons, but not in the pyramidal neurons of the PRC.

Concerning voltage-dependence of resonance, the analysis of F_res_ at different membrane potential ranging from −90 to −55 mV showed very similar values. However, the characterization of the resonance strength at different holding membrane potentials, between −90 and −55 mV, indicated that the resonant neurons manifest a voltage-dependent resonance. In pyramidal neurons the *Q*-value exhibited a peak at around −70 mV, whereas in interneurons the maximum *Q*-value was measured between −80 and −70 mV. These observations indicated a prominent response at potential value at or just below resting membrane potential. The abolishment of resonance in PRC neurons following I_h_ current block with ZD7288 suggests that the expression of the HCN channels is mandatory for the definition of the resonant mechanism in PRC in both groups of neurons tested, confirming the important role played by HCN in the subthreshold resonance, as already demonstrated for other neocortical (Hutcheon et al., 1996) and limbic areas (Shay et al., 2012). The expression of this conductance only in resonant cells could also justify the more depolarized membrane resting potential of resonant compared to non-resonant neurons. Interestingly, the application of TTx caused a decrease in the resonant peak, but did not abolish the resonant behavior, suggesting that in pyramidal neurons of the PRC a persistent sodium current is able to amplify the resonance produced by other conductances, as demonstrated in other brain areas. Indeed, at membrane potentials ranging between −70 and −60 mV the persistent sodium current produced in pyramidal neurons a significant amplification of the resonant peak that became steeper and sharpened, whereas the F_res_ was not affected by the application of TTx. By contrast, in interneurons the amplification of the resonance mediated by persistent sodium current was not observed, probably due to a lack or to a low density of I_Na__P_ channels in these cells, as shown in CA1 (Hedrich et al., 2014) and in neocortex (Aracri et al., 2006). Alternatively, it could be hypothesized, in the case of interneurons, a concurrent activation of persistent sodium current together with a current, such as the potassium inward rectifier, that attenuates resonance. The experimental data were replicated by using a MatLab model, containing I_leak_, I_h_ and I_NaP_ channels. Currents’ kinetics and gating properties were derived by literature (Hodgkin and Huxley, 1952; Spain et al., 1987), due to the lack of data describing these properties in the PRC pyramidal and GABAergic neurons. To overcome the limitations of the model and for a future upgrade, these parameters could be derived directly from PRC neurons. However, since these electrophysiological parameters are quite conserved (Zhou and Lipsius, 1992; Kiss, 2008; Kase and Imoto, 2012), we believe that reliable results could be obtained also by using general equations. Indeed, our computational model faithfully reproduced the experimental behavior, confirming the indispensable role of I_h_ in the genesis of resonance and the involvement of the persistent sodium current in the amplification of the resonance peak. Furthermore, it is important to consider that temperature strongly influences the F_res_ and this happens also in PRC, as predicted by our computational model and confirmed by our experimental data. We found that the rise of temperature from 23 to 36°C significantly increased the F_res_ of both pyramidal neurons and interneurons, aligning these data to the well-known resonance frequency already observed in the brain areas functionally linked to the PRC.

The functional behavior of the individual neurons affects and defines the processing of information within neural circuits. Membrane resonance at a given frequency has already been described in many other cells across different brain areas and is strictly dependent on intrinsic membrane properties, synaptic inputs, and modulatory effects. The interaction between the passive properties of the cell and active conductances mediated by ion channels define not only membrane resonance but also action potential timing, synaptic integration, and membrane potential oscillations. Altogether these properties enable neurons to react preferentially to synaptic inputs at specific frequencies and also influence the dynamics of the neural networks of which they are part. The inhibition produced by interneurons on pyramidal neurons and other interneurons is critical for the synchronization of neural activity (Mann and Paulsen, 2007). In the PRC the resonance of interneurons at 1–3 Hz suggests that synaptic inputs at these frequencies will likely entrain the entire neuronal network of the PRC to oscillate at the range of 1–3 Hz, typical of the delta rhythm during non REM sleep.

Coherent network oscillations distributed in different brain regions are critical to achieve learning and memory and in general to process neural information. Low frequency oscillations are usually engaged to connect different cerebral regions to transfer or retrieve distributed information. On the other hand, coherent high frequency oscillations are more restricted to localized neuronal networks and seem to be involved in encoding of sensory information, in promoting synaptic plasticity (Fries, 2005), and in the definition of the sequence of information transfer (Sejnowski and Paulsen, 2006; Fries et al., 2007).

Semantic memories are stored in different cortical areas and a still unanswered question is how these distributed memories are recalled and linked together to obtain an object representation. In the PRC, the representation of the objects learned throughout experience-dependent episodes are associated with their memorized features. The retrieving of these associations is based on the information exchange among the PRC and distributed cortical areas. The PRC, hence, via its afferent and efferent connections acts as a hub of semantic memory.

Slow frequency oscillations (0.5–4 Hz) that are widely described in different areas linked to PRC such as the neocortex, amygdala, and lateral EC (Collins et al., 2001) could subserve the pivotal role, played by the PRC, for binding the information stored sparsely in different cortical areas to obtain a coherent neural representation to be sent to the hippocampus. Similarly, but in opposite direction, short-term memory developed in hippocampus should be forwarded and stored in distributed neocortical regions throughout a consolidation process that likely use the same PRC hub and oscillatory mechanisms. The reverberating activity between neocortex and hippocampus, especially during slow wave sleep, indeed represents the mechanism by which the brain promotes the redistribution and strengthening of memory representation in cortical regions (Klinzing et al., 2019). Another aspect which worth to be considered is related with dendritic resonance. Detailed studies have characterized the spatial properties of the resonance along the dendrite-to-soma extension in hippocampal (Narayanan and Johnston, 2007; Hu et al., 2009) and prefrontal (Kalmbach et al., 2013) pyramidal neurons. In particular, recordings performed on CA1 pyramidal neurons showed that membrane resonance frequency could differ threefold between soma and apical dendrites. These results suggest that CA1 pyramidal cells could act as stimulus-tuned filters for spatially separated synaptic inputs. The cognitive processes of the PRC strongly rely on the presence of separated synaptic inputs from piriform, entorhinal and neocortical cortices, and amygdala to the PRC and its ability to integrate them. Then, future experiments should also be addressed to investigate the resonance properties along the soma-to-dendrite extension in pyramidal neurons of the PRC.

## Data Availability Statement

The raw data supporting the conclusions of this article will be made available by the authors, without undue reservation.

## Ethics Statement

The animal study was reviewed and approved by the National Ministry of Health and Local Ethical Committee of the University of Pavia (OPBA).

## Author Contributions

NB, FT, PS, and GB conceived the study and designed the experiments. NB, FT, PS, CM, FR, NM, and AC performed the *in vitro* experiments and data analysis. MP, SR, and MT defined and performed the computational model. YY developed and provided the GAD67-GFP mice. CD edited the manuscript. NB, FT, MT, CM, and GB wrote the manuscript. GB supervised the study. All authors contributed to the article and approved the submitted version.

## Conflict of Interest

The authors declare that the research was conducted in the absence of any commercial or financial relationships that could be construed as a potential conflict of interest.
